# Sucralfate and Lidocain: Antacid 50:50 solution in Post Esophageal Variceal Band Ligation Pain

**DOI:** 10.12669/pjms.324.9645

**Published:** 2016

**Authors:** Muhammad Hafeez, Ehsan Kadir, Anjum Aijaz

**Affiliations:** 1 Dr. Muhammad Hafeez, FCPS(Med), FCPS(Gastro) FACG, MASGE(USA), FRSPH, SCE RCP Gastro(UK); 2 Assistant Professor of Medicine and Gastroenterologist, Combined Military Hospital Kharian Cantt Pakistan; 3 Dr. Ehsan Kadir, M.Sc Nutrition Assistant Professor of Community Medicine, Combined Military Hospital Multan Pakistan; 4 Dr. Anjum Aijaz, MBBS, Dip Derm(UK). Consultant Dermatologist, Combined Military Hospital Malir Cantt Pakistan

**Keywords:** EVBL, Post EVBL pain, Sucralfate, Lidocain: antacid

## Abstract

**Objective:**

To compare the effectiveness of pain relief of Sucralfate and lidocain antacid 50:50 solution in post esophageal variceal band ligation pain.

**Methods:**

All patients who had under gone Esophageal Variceal Band Ligation (EVBL) were included in the study. Patients un-willing to be included in the study or those who didn’t have post EVBL pain were excluded. Patients with post EVBL pains were divided into two groups: one group was given sucralfate and other was given lidocaine: antacid 50:50 solution. Both were inquired about the duration of the pain relief after the medication. The results were analyzed on SPSS 23. Independent samples T-test was performed to find out whether the difference in duration of pain relief was significantly different in the two groups

**Results:**

Out of 110 patients who have EVBL, 66(60.00%) had pain and 44(40.00%) were pain free. In the pain group 46 (69.7%) were given sucralfate and 20 (30.3%) were given lidocain: antacid 50:50 solution. Mean duration of pain relief in two groups was 2.78 (SD ± 2.096) and 2.5 days (SD ±. 0.76) respectively. Independent samples T-test results revealed that there was no statistically significant difference in the duration of pain relief between these two groups with p value 0.426.

**Conclusion:**

Both Sucralfate and Lidocain: antacid 50:50 solutions are effective in relieving the post EVBL pain. However, no statistically significant difference in duration of pain relief was detected in separate groups of patients treated with either treatment.

## INTRODUCTION

Upper Gastrointestinal bleed secondary to esophageal varices is a life threatening situation in Decompensated Chronic Liver Disease (DCLD). With single bleed mortality increased to 20%, rebleed in 40% in six weeks’ time and 75% at one year.^1^ Injection sclerotherapy revolutionized the treatment and remain only successful mode of therapy for many years.^2^ Then question rose because of procedure related complications like rebleeding from large ulcers, stricture formation especially when performed with lesser interval.^3^ It was replaced with Esophageal Variceal band ligation because of lesser complications like transient dysphagia, small ulcers and less risk of rebleed.^4^,^5^

Post Esophageal Variceal band ligation pain is a common complication, mostly it is mild to moderate in intensity but may be severe in few cases. Usually it settles itself but may require some intervention to relieve it. Esophageal pain is due to stimulation by chemoreceptors due to acid or hyperosmolar substances, mechano-receptors by distension or thermos-receptors by hot and cold food.^6^ Sucralfate is sucrose sulfate-aluminum complex that in pH less than 4 reacts with Hydrochloric acid and make a paste like material and cover the rough or ulcerated surface and act as acid buffer.^7^ Antacids are effective in relieving dyspepsia, addition of lidocain increase efficacy and has additional local anesthetic and anti-spasmodic effect.^8^ Keeping in view two medicines were tried in this study i.e. sucralfate and lidocain: antacid 50:50 solution to find out the effects to relieve the pain.

## METHODS

This study was carried out in combined Military Hospital Kharian that is a tertiary care hospital. Study was carried out from Dec 2014 to September 2015. It was prospective therapeutical trial (interventional cohort). Study was approved by ethical committee of the Hospital. Non-probability, purposeful, criterion sampling was done. After the informed consent, procedure of upper G I endoscopy and EVBL was explained. Throat spray with lignocaine 4% solution and conscious sedation with injection midazolam was done. Dose of Midazolam was adjusted keeping underlying disease and age of the patient in mind. Upper GI endoscopy with high definition video endoscope system was performed. Multi-band ligators manufactured by the Wilson-Cook Medical GI endoscopy company were used.

All patients who had undergone esophageal variceal band ligation and had post EVBL pain were included and those unwilling to be included or didn’t have pain were excluded. Patients were inquired about the pain as per numeric pain rating scale (N-11).^9^ it is 11 point scale to assess the severity. Its severity was classified as none, mild, moderate and severe for points 0, 1-3, 4-6 and 7-10 respectively. Patients with post EVBL pain were randomly divided into two groups by the Gastroenterology medical team. One group was given sucralfate 15 ml and second group was given 15 ml 4% lidocaine: antacid 50:50 solution thrice daily one hour before meals and before going to bed. As lidocain: antacid 50:50 solution was not available commercially. It was prepared in hospital pharmacy under the supervision of qualified Pharmacist. It was stored in room temperature protected from heat and freezing. Freshly prepared solution was consumed in one week time. Patients once experienced pain was the start / zero time, and as per random allocation, were given the above mentioned medicine in two separate groups. Patients were asked periodically about the relief of pain. It was recorded in days once patients were pain free. Patients were discharged from the hospital as they were pain free with follow up upper G I Endoscopy after two weeks. SPSS version 23 was used for statistical analysis. Descriptive statistics in terms of mean ± standard deviation were calculated for continuous variables while frequencies and percentages were calculated for categorical variables. Independent samples t-test was applied to the mean of the pain relief duration in the two randomly distributed groups receiving different treatments.

## RESULTS

Out of 110 patients with EVBL, 66(60%) experienced post EVBL pain and were selected for the study. Out of the selected 66 patients, 45 (68.2%) were males and 21 (31.8%) females. Minimum age was 12 and maximum 82 years with mean of 53 and SD ± 13.41. Male to female ratio was 2.3:1. Frequencies of Child classes A, B & C of the patients were 35%, 60% and 15% respectively. Out of 66 patients with post-EVBL pain 15 (22.7%) had mild, 43 (65.2%) moderate and 8 (12.1%) severe pain after esophageal variceal band ligation. Main reason for endoscopy in these cases was upper G I bleed followed by screening for esophageal varices and surveillance of EVBL.

In the initial 110 patients before case selection total of 255 banding sessions were done, minimum one and maximum of 10 with SD ± 1.31. Mode calculated for the number of sessions was two. 660 bands were applied with average of 6 per session. The selected 66 patients which were randomly divided into two groups, first group 46 (69.7%) were given sucralfate; their response in number of days is shown in Table-I. Earliest response was in one day and maximum in 10 days with mean of 2.78 with SD ± 2.096 days and median 2 days. Mode of pain response in no of days was 2 days seen in 26 (56.5%) cases, followed by next frequent response time of 3 days seen in 7(15.2%) cases. In second group twenty patients (30.3%) were given lidocain: antacid 50:50 solution.

**Table-I:** Response to pain relief (number of days) in Sucralfate group.

Response in no of days	Frequency	Percentage %	Cumulative Percentage%
1	6	13.04	13.04
2	26	56.52	69.56
3	7	15.21	84.78
4	2	4.34	89.13
6	1	2.17	91.30
7	1	2.17	93.47
8	1	2.17	95.65
10	2	4.34	100.00

Total	46	100%	

Their response distribution in number of days is presented in Table-II. Earliest response was in one day and maximum in four days with mean of 2.5 days with SD ±. 0.76 and median two days. Mode of second group was two days seen in 13 (65%) cases. Pain relief response in the two groups as measured in number of days after sucralfate and lidocain:antacid solution administration was analyzed by Independent samples T-test, P value of <0.05 was considered statistically significant. In both medicines duration of response mode calculated was within two days i.e., 56.5% and 65.0% in sucralfate and lidocain: antacid 50:50 solution respectively and signifies that both medicines are effective in controlling post EVBL pain.

**Table-II:** Response to pain relief (number of days) in Lidocain: antacid group.

Response in no of days	Frequency	Percentage %	Cumulative Percentage %
2	13	65.00	65.00
3	4	20.00	85.00
4	3	15.00	100.00

Total	20	100.00%	

SPSS version 23 was used for statistical analysis. Independent samples T test was applied at 95% significance level. Keeping “number of days for pain relief response” as independent variable, “type of treatment administered” was selected as the grouping variable. Results were interpreted as follows. T-test for differences in mean between two groups was not significant with p value 0.426. Therefore, no statistically significant difference in duration of pain relief was observed between the two groups.

## DISCUSSION

Pharmacological and EVBL is recommended mode of treatment for the control of esophageal variceal bleed.^10^ EVBL is preferred treatment when compared with sclerotherapy with fewer side effects.^11^ Post EVBL pain is still a common complication, mostly it is mild to moderate in intensity as observed in the present study i.e. 87.9%, and severe in 12.1%. Frequency of pain 66 (60%) is almost the same as observed by Ali et al. in which the frequency of pain in multiple versus single band therapy groups was 30% and 82% respectively.^12^ Patients with moderate to severe cases required some intervention to relieve the pain. Sucralfate is effective by protective adherence to denuded surfaces^13^ and gives a quick pain relief as early as two days as we observed in maximum number of patients in 26 (56.5%). Sucralfate is found effective in this study as observed in other type of esophageal injuries.^14^,^15^ Immediate Post EVBL pain is because of esophageal spasm. Gastrointestinal cocktail containing antacid and lidocain is effective in relieving the symptoms owing to its anti-spasmodic and local anesthetic effects.^16^

In the first group 32 (69.56%) patients were pain free in on 2^nd^ day of post EVBL pain and 41(89.13%) by 4^th^ day. In the 2^nd^ group 13 (65.00%) patients were asymptomatic on 2^nd^ and 100% on 4^th^ day. It points to a quicker and sustained relief in second group on 2^nd^ day and 4^th^ day. 2(5%) patients had response on 10^th^ day in first group and no patient was symptomatic beyond 4^th^ day in the second group that again points more effectivity in lidocain: antacid group. Confounding factor being that the second group was smaller than the 1^st^ one. Ulcers form once the band slough off at post esophageal variceal band ligation site leads to stricture formation and obliterates varices. Ulcers are superficial, resolve faster as compared to sclerotherapy induced ulcers i.e., 14 versus 21 days.^17^,^18^ Pain is usually less in severity in the ulcer stage because of superficial ulceration in EVBL; only 15% had mild to moderate pain in our study. Sucralfate and lidocain antacid 50:50% both were equally effective in this stage too. However it may become difficult to predict exact response in different genders because of diverse sensitivity in different patients observed by Hobson et al.^19^ in our study pain ratio in male to female was 2.3:1. Mean duration of symptoms was 64.73 hours with SD ± 43.12 hours in comparison to a study by Hou et al.^20^ mean duration being 8.27±5.52 and 9.55±5.82 hours respectively in two groups of patients. It necessitates the importance of requirement of medication to relief the pain.

In the current study, the number of enrolled patients was good enough and study continued for ten months, still there were certain limitations. It was a single center based study and secondly GI cocktail was not easily available and had to be prepared in the hospital Pharmacy. Keeping effectivity of this medicine in post EVBL pain in mind its availability can be stressed upon.

## CONCLUSION

Both Sucralfate and lidocain: antacid solutions are effective in post EVBL pain relief. There was no statistical difference in mean pain relief duration between these two groups. However second group has slight edge in early and sustained response. More studies are required to strengthen the findings.
